# Convergence and Divergence of Signaling Events in Guard Cells during Stomatal Closure by Plant Hormones or Microbial Elicitors

**DOI:** 10.3389/fpls.2016.01332

**Published:** 2016-08-24

**Authors:** Srinivas Agurla, Agepati S. Raghavendra

**Affiliations:** Department of Plant Sciences, School of Life Sciences, University of HyderabadHyderabad, India

**Keywords:** ABA, cytosolic free Ca^2+^, cytosolic pH, ROS, guard cells, ion channels, nitric oxide, secondary messengers

## Abstract

Dynamic regulation of stomatal aperture is essential for plants to optimize water use and CO_2_ uptake. Stomatal opening or closure is accompanied by the modulation of guard cell turgor. Among the events leading to stomatal closure by plant hormones or microbial elicitors, three signaling components stand out as the major converging points. These are reactive oxygen species (ROS), cytosolic free Ca^2+^, and ion channels. Once formed, the ROS and free Ca^2+^ of guard cells regulate both downstream and upstream events. A major influence of ROS is to increase the levels of NO and cytosolic free Ca^2+^ in guard cells. Although the rise in NO is an important event during stomatal closure, the available evidences do not support the description of NO as the point of convergence. The rise in ROS and NO would cause an increase of free Ca^2+^ and modulate ion channels, through a network of events, in such a way that the guard cells lose K^+^/Cl^−^/anions. The efflux of these ions decreases the turgor of guard cells and leads to stomatal closure. Thus, ROS, NO, and cytosolic free Ca^2+^ act as points of divergence. The other guard cell components, which are modulated during stomatal closure are G-proteins, cytosolic pH, phospholipids, and sphingolipids. However, the current information on the role of these components is not convincing so as to assign them as the points of convergence or divergence. The interrelationships and interactions of ROS, NO, cytosolic pH, and free Ca^2+^ are quite complex and need further detailed examination. Our review is an attempt to critically assess the current status of information on guard cells, while emphasizing the convergence and divergence of signaling components during stomatal closure. The existing gaps in our knowledge are identified to stimulate further research.

## Introduction

Stomata are tiny pores found on the leaf surface of higher plants, which facilitate the evaporation of H_2_O via transpiration and intake of CO_2_ for photosynthetic carbon assimilation (Acharya and Assmann, 2009). Stomata are also major points of entry for pathogens into the plants (Melotto et al., 2006, 2008). Therefore, the regulation of stomatal aperture is essential for limiting the loss of H_2_O as well as restricting pathogen entry. The guard cells are quite sensitive to several internal and external stimuli, including abiotic (drought, light, temperature, high CO_2_, humidity) or biotic factors (pathogens and elicitors). Plant hormones (such as abscisic acid, ABA, methyl jasmonate, MJ) and polyamines (PAs) induce stomatal closure. Elicitors such as salicylic acid (SA), chitosan, and Flg22 also cause stomatal closure (Alcázar et al., 2010; Jing et al., 2012; Gayatri et al., 2013; Ye et al., 2013; Agurla et al., 2014). Stomata open when guard cells are turgid and close when the guard cells are flaccid (Blatt, 2000). During stomatal opening, guard cells accumulate osmotically active components, such as potassium ions, anions, malate and sucrose, leading a decrease in water potential, influx of water, and increase in turgor. In contrast, the reversal of these events leads to flaccidity in guard cells and stomatal closure (Vavasseur and Raghavendra, 2005; Bright et al., 2006; Roelfsema et al., 2012).

Among several effectors, the effects of ABA (a phytohormone) on stomatal movements have been studied in detail. ABA induced stomatal closure is mediated by many signaling components like cytoplasmic pH, reactive oxygen species (ROS), reactive nitrogen species (nitric oxide, NO), cytosolic free Ca^2+^, G-proteins, protein kinases, protein phosphatases, phospholipids, phospholipases, and sphingolipids (Wang and Song, 2008; Raghavendra et al., 2010; Umezawa et al., 2010; García-Mata and Lamattina, 2013; Song et al., 2014). The diverse spectrum of signaling components during stomatal closure have been reviewed frequently (Kim et al., 2010; Joshi-Saha et al., 2011; Gayatri et al., 2013; Agurla et al., 2014; Kollist et al., 2014; Song et al., 2014; Murata et al., 2015; Lee et al., 2016).

There are yet questions about the sequence of the signaling events during stomatal closure. For e.g., cytosolic free Ca^2+^ may act at either downstream or upstream of ROS/NO. The changes in cytosolic pH of guard cells may be important at either downstream or upstream of ROS or NO. The production of NO precedes that of ROS, but NO can act as antioxidant as well. Despite these ambiguities, it is clear that a rise in ROS or NO triggers a rise in free Ca^2+^ of guard cells, modulate the ion channels and cause an efflux of K^+^/Cl^−^/malate, leading to loss in turgor of guard cells. We emphasize that the signaling events during stomatal closure converge at ROS, cytosolic Ca^2+^, and ion channels. Similarly, ROS, NO, and Ca^2+^ form the points of divergence.

### Points of convergence: ROS, cytosolic free Ca^2+^, and ion channels

**Figure 1:**
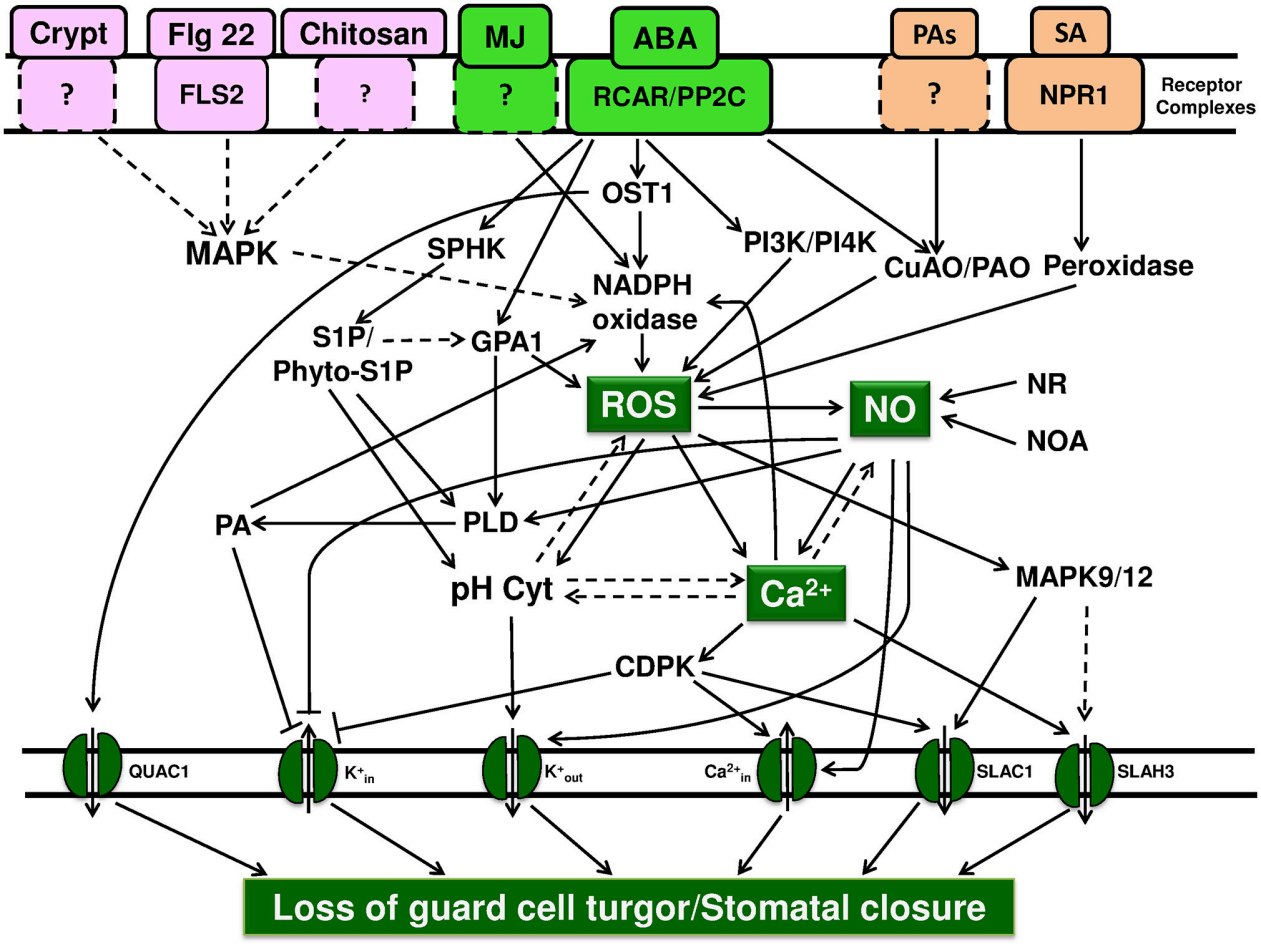
**Key points of convergence and divergence during stomatal closure in response to plant hormones and elicitors**. Stomatal closure is the result of ion efflux out of guard cells, loss of their turgor, and forms the ultimate step during signal transduction. We suggest that ROS, cytosolic free Ca^2+^, and ion channels form points of convergence during stomatal closure by a variety of abiotic/abiotic factors. Similarly, ROS, NO, and cytosolic Ca^2+^ are identified as points of divergence. The activation of NADPH oxidase and ROS production are among the earliest events. Similarly, the modulation of ion channels, influx of free Ca^2+^ along with efflux of K^+^ and anions, are the final steps, leading to the loss of ions/turgor of guard cells. The binding of ABA to RCAR/PYR or Flg22 to FLS2 or SA to S-receptor are well established, while receptors of cryptogein, chitosan, and PAs are yet to be characterized. ROS: When ABA binds to the receptor (RCAR/PYR/PYL), PP2C becomes non-functional, leading to phosphorylation, and activation of OST1 protein kinase. The elevated kinase activity along with Ca^2+^, activates NADPH oxidase, and subsequently elevates ROS production. Besides NADPH oxidase, CuAO/PAO are also involved in the increase of ROS in guard cells. The levels of ROS can be elevated by also peroxidase, for e.g., upon salicylic acid binding to its receptor. Further, G-protein alpha subunit induces the ROS production through the activation of NADPH oxidase. Modulation of ROS levels by NO, cytosolic Ca^2+^, cytosolic pH can occur by direct or indirect mechanisms but these reactions need to be established. Cytosolic free Ca^2+^: the rise in the levels of ROS and NO, can increase the levels of cytosolic free Ca^2+^, by either release of Ca^2+^from internal stores or influx of external Ca^2+^ through plasma membrane ${\text{Ca}}_{\text{in}}^{2+}$channels. Ca^2+^ also activates SLAH3 and SLAC1 ion channels, while inhibiting ${\text{K}}_{\text{in}}^{+}$ ion channels. Ion channels: the modulation of cation/anion channels results in the net efflux of K^+^/Cl^−^/ malate and influx of Ca^2+^, making guard cells to lose turgor and causing stomatal closure. NO: NR, nitrate reductase; NOA, nitric oxide associated 1 are the sources of NO. Although there are suggestions that ROS, cytosolic Ca^2+^ or cytosolic pH can elevate NO levels, the mechanism is not known. The rise in NO leads to divergent actions, namely the rise in cytosolic Ca^2+^, activation of PLD, and subsequently NADPH oxidase. Further, NO activates ${\text{K}}_{\text{out}}^{+}$ ion channels, inhibits K^+^ channels, and activates ${\text{Ca}}_{\text{in}}^{2+}$ ion channels. Other components: The role of cytosolic pH is not completely understood. The available evidence suggests that the cytosolic pH may act parallely with the events involving ROS/NO/cytosolic free Ca^2+^. Similarly, G-proteins, phospholipids, phospholipases, phosphatidyl inositol kinases, sphingolipids, and MAP kinases also act in such a way to cause the loss of turgor in guard cells and stomatal closure. Solid arrows represent the events which are documented, while broken arrows represent the possible effects/suggestions.

When guard cells are exposed to signals originating from abiotic or biotic factors the process of signal transduction is initiated. During this process, three points can be recognized as those of convergence: ROS, cytosolic free Ca^2+^, and anion channels. For e.g., plant hormones (such as ABA or MJ) and microbial elicitors invariably cause an increase in the levels of ROS or NO in guard cells, leading to rise in free Ca^2+^ within the guard cells (Table 1). There are excellent reviews, emphasizing the role of ROS (Kollist et al., 2014; Song et al., 2014; Murata et al., 2015), NO (Hancock et al., 2011; García-Mata and Lamattina, 2013; Gayatri et al., 2013; Agurla et al., 2014), and cytosolic free Ca^2+^ in guard cells (Kim et al., 2010; Roelfsema and Hedrich, 2010). Hormones and elicitors interact with different receptor entities, but the subsequent steps converge to activate NADPH oxidase, increase ROS, NO, and Ca^2+^ in guard cells (Figure 1). Although NO in guard cells is a key signaling component, there is no sufficient evidence to describe it as point of convergence. While it is clear that ROS can cause an increase in NO of guard cells, no other components that can raise NO levels has been described.

**Table 1:** **Major points of convergence as well as divergence during signal transduction leading to stomatal closure by hormones or elicitors**.

**Convergence**	**Upstream component**	**References**
**ROS**		
	NADPH oxidase	Kwak et al., 2003
	Peroxidase	Khokon et al., 2010
	Copper amine oxidase	An et al., 2008
	G-protein alpha subunit (GPA)	Zhang et al., 2011
	OST1 protein kinase	Mustilli et al., 2002
	Cytosolic free Ca^2+^	Kobayashi et al., 2007
	Phosphatidic acid	Zhang et al., 2004
	MAPK	Meng and Zhang, 2013
	PI3K/PI4K	Park et al., 2003
	S1P	Ma et al., 2012
	PA/ Phospholipase Dα1	Zhang Y. et al., 2009
	Cytosolic pH	Suhita et al., 2004
**CYTOSOLIC FREE Ca^2+^**		
	ROS	Pei et al., 2000
	NO	Hossain et al., 2014
	Inositol 1,4,5-trisphosphate	Gilroy et al., 1990
	Cyclic ADP ribose	Leckie et al., 1998
	Calcineurin-B like proteins	Drerup et al., 2013
**ION CHANNELS**		
**${\text{Ca}}_{\text{in}}^{2+}$ channels**		
	Ca^2+^	Mori et al., 2006
	NO	Garcia-Mata et al., 2003
**Inward-rectifying K^+^ channels (KAT1)**		
	PA	Uraji et al., 2012
	Cytosolic free Ca^2+^	Grabov and Blatt, 1999
	NO	Sokolovski and Blatt, 2004
**Outward rectifying K^+^ channel (GORK)**		
	pH	Hosy et al., 2003
	Cytosolic free Ca^2+^	Pei et al., 1998
	NO	Sokolovski and Blatt, 2004
**Slow anion channel 1 (SLAC1)**		
	MAPK9/12	Danquah et al., 2014
	Cytosolic free Ca^2+^	Geiger et al., 2010
**Slow anion channel Homolog 3 (SLAH3)**		
	Cytosolic free Ca^2+^	Geiger et al., 2010
**Quick anion channels (QUAC1/ALMT6)**		
	OST1	Engineer et al., 2016
**Divergence**	**Downstream component**	**References**
**ROS**		
	NO	Bright et al., 2006
	MAPK9/12	Jammes et al., 2009
	Cytosolic free Ca^2+^	Pei et al., 2000
	Cytosolic pH	Zhang et al., 2001
**NO**		
	PLDδ	Distéfano et al., 2012
	Cytosolic free Ca^2+^	Zhao et al., 2013
	Cytosolic free ${\text{Ca}}_{\text{in}}^{2+}$	Garcia-Mata et al., 2003
	${\text{K}}_{\text{in}}^{+}$channels	Garcia-Mata et al., 2003
	${\text{K}}_{\text{out}}^{+}$ channels	Sokolovski and Blatt, 2004
**CYTOSOLIC FREE Ca^2+^**		
	NADPH oxidase	Kimura et al., 2012
	NO	Garcia-Mata and Lamattina, 2007
	Cytosolic pH	Islam et al., 2010
	SLAC1	Laanemets et al., 2013
	SLAH3	Geiger et al., 2011

The convergence is illustrated by the multiple upstream elements leading to an increase in the given component. Similarly, the divergence occurs when multiple components are modulated by the given signaling element. An illustration is given in Figure 1.

ROS, reactive oxygen species; NO, nitric oxide; MAPK, mitogen-activated protein kinases; SLAC1, slow anion channel-associated 1; SLAH3, slow anion channel homolog 3; Ca^2+^, calcium; H_2_O_2_, hydrogen peroxide; K_in_ channel, K^+^ inward rectifying channel; K_out_ channel, K^+^ outward rectifying channel; PA, phosphatidic acid; OST1, open stomata 1; QUAC1, quick anion channel 1; ALMT, aluminum activated malate transporters; PLD, phospholipase D; S1P, sphingosine-1-phosphate.

#### Reactive oxygen species (ROS)

A marked rise in ROS of guard cells is a consistent feature of stomatal closure induced by ABA, MJ, and even microbial elicitors (Zhang H. et al., 2009; Song et al., 2014). While the effect of ABA on NADPH oxidase is mediated by ABA-receptors-protein phosphatase interactions (Raghavendra et al., 2010), the mechanism of NADPH oxidase stimulation by elicitors is ambiguous. Certain MAP kinases activated by elicitors could in turn activate NADPH oxidase (Zhang H. et al., 2009).

There has been overwhelming evidence that NADPH oxidase is the major ROS source in ABA, MJ, or SA induced stomatal closure. However, the source of ROS may not always be NADPH oxidase, as ROS production in response to elicitors, such as SA, yeast elicitor, and chitosan can occur through a salicylhydroxamic acid (SHAM) sensitive peroxidase or amine oxidases (e.g., copper amine oxidase or polyamine oxidase) (Khokon et al., 2011; Gao et al., 2013; Murata et al., 2015). During stomatal closure induced by methylglyoxal (MG), isothiocyanates or thiocyanates, the rise in ROS of *Arabidopsis* guard cells was mediated by a SHAM sensitive peroxidase (Hoque et al., 2012; Hossain et al., 2013). Activation of NADPH oxidase can occur also by phosphatidic acid (PA) (Zhang H. et al., 2009). Thus, the ROS of guard cells is a major point of convergence. The ROS production by different systems, involving NADPH oxidase or peroxidase has been reviewed recently by Murata et al. (2015).

#### Cytosolic free calcium

Calcium (Ca^2+^) is an important secondary messenger during stomatal closure (McAinsh et al., 1990; Hubbard et al., 2012). The role of Ca^2+^ is confirmed by monitoring of Ca^2+^ in guard cells by fluorescent probes, the Ca^2+^ chelators, and Ca^2+^ channel blockers (Pei et al., 2000; Kim et al., 2010). The rise in Ca^2+^, due to influx or release from internal sources like endoplasmic reticulum, further activates anion channels and inhibits the ${\text{K}}_{\text{in}}^{+}$ channels, all leading to stomatal closure. There are suggestions that Ca^2+^ may act also upstream of ROS and NO (Garcia-Mata et al., 2003). In contrast, Zhang et al. (2011) observed that calcium channels functioned downstream of H_2_O_2_ in G-protein α-subunit (*gpa1*) mutants. In *gpa1* mutants, ABA-induced ROS production was disrupted, but Ca^2+^ channels were activated by exogenous H_2_O_2_ application.

#### Ion channels

The ion channels represent the last step of signal transduction, leading to stomatal closure. The ionic status driven by the activity of cation/anion channels determines the turgor state of guard cells. Rise in free Ca^2+^ of guard cells causes the efflux of K^+^/Cl^−^/other ions. The detailed descriptions of ion channels, their intracellular location, encoding genes, along with mutants are made in a few reviews (Hedrich, 2012; Roelfsema et al., 2012; Kollist et al., 2014). Plants have several types of K^+^ channels, which can allow either inward or outward movement of K^+^. The ${\text{K}}_{\text{in}}^{+}$ channels open up, when the membrane potential becomes hyperpolarized. In contrast, outward-rectifying K^+^channels (${\text{K}}_{\text{out}}^{+}$) open when the membrane potential is depolarized.

Guard cell Ca^2+^-permeable cation channels are stimulated by H_2_O_2_ and NO, whose levels are raised by ABA or MJ during stomatal closure (Mori et al., 2006; Rienmüller et al., 2010). Elevated free Ca^2+^ in guard cells can be due to the activation of Ca^2+^ channels in not only plasma membrane but also vacuolar or internal membrane network. The activation of ion channels would promote efflux of malate and other anions make the guard cells lose turgor and cause stomatal closure. But, there is considerable ambiguity on the relative dominance and specificity of different ion channels. Guard cells are known to contain slow anion channel-associated 1 (SLAC), quick anion channel 1 (QUAC), slow anion channel homolog 3 (SLAH), and even aluminum activated malate transporters (ALMT) (Roelfsema et al., 2012). Further work is required to elucidate the role of each of these different types of anion channels and their interactions.

### Points of divergence: ROS, NO, and cytosolic free Ca^2+^

The rise in levels of ROS, NO, or cytosolic free Ca^2+^ in guard cells trigger multiple events downstream (Table 1). The ability to induce diverse effects makes these three signaling components qualified to be the points of divergence (Figure 1). The rise in ROS of guard cells initiates several downstream events: NO production, elevation of cytosolic free Ca^2+^, and rise in cytosolic pH (Wang and Song, 2008; Song et al., 2014). Kinetic studies indicated that ROS production was prior to the NO production (Gonugunta et al., 2008). The positioning of the ROS was further confirmed by using Arabidopsis mutants and hydrogen-rich water (HRW) (Xie et al., 2014). The impaired NO synthesis and stomatal closure in response by HRW and rescue of closure by exogenous application of NO in *rbohF* mutant indicated that ROS functioned as an upstream signaling component. The importance of ROS in NO production was also demonstrated in mutants deficient in G-proteins and nitrate reductase (Bright et al., 2006; He et al., 2013).

Nitric oxide (NO) is a small, gaseous molecule involved in growth, development and even disease resistance of plants (Domingos et al., 2015). Studies using modulators (scavengers/inhibitors/donors) of NO production emphasized the importance of NO during stomatal closure (Gayatri et al., 2013; Agurla et al., 2014). NO production in guard cells of *Arabidopsis* and *Vicia faba* is essential for stomatal closure by SA and yeast elicitor (Sun et al., 2010; Khokon et al., 2011). Real time monitoring studies suggested that NO acted as a downstream signaling component to the ROS as well as pH (Gonugunta et al., 2008; Srivastava et al., 2009). Nitric oxide synthase (NOS) is the source of NO in animal cells, but the presence/operation of NOS in plant cells is quite uncertain. Both nitrate reductase (NR) and NOA1 (nitric oxide associated) are shown to be the sources of NO in guard cells of *V. faba* and Arabidopsis (Hao et al., 2010; Gao et al., 2013).

The interaction of NO with the other signaling components is quite crucial (Gayatri et al., 2013). In guard cells, NO can cause multiple effects, namely rise in internal Ca^2+^, cytosolic alkalization, and activation of ${\text{K}}_{\text{out}}^{+}$ channels (Gonugunta et al., 2008; Jing et al., 2010). NO is also essential for the elevation of the signaling components, like PLDα1 and PLDδ, during PA induced stomatal closure (Distéfano et al., 2008, 2010; Uraji et al., 2012).

The components of downstream signaling by Ca^2+^ in guard cells are quite intriguing. The changes in Ca^2+^ are sensed and mediated by the different types of intracellular calcium binding proteins like calmodulins, calcium dependent protein kinases (CDPKs, particularly, CPK3, and CPK6) and calcium sensing receptors (CAS) (Mori et al., 2006). Ca^2+^-dependent CPK6, CPK21, and CPK23 activate SLAC1 in oocytes (Geiger et al., 2010; Brandt et al., 2012). In contrast, Ca^2+^-independent protein kinases like OST1 are involved in ABA activation of intracellular calcium channels (Murata et al., 2015). Ca^2+^-independent SnRK2 protein kinases such as OST1, have been shown to activate SLAC1 in *Xenopus leavis* oocytes (Geiger et al., 2009; Lee et al., 2009; Brandt et al., 2012). Such Ca^2+^ activation of S-type anion currents is an early and essential step during stomatal closure (Siegel et al., 2009; Chen et al., 2010).

### Other components

#### Cytosolic pH

Cytoplasmic pH is a signaling component in developmental processes, such as root growth (Scott and Allen, 1999). A marked rise in cytoplasmic pH is a common feature during stomatal closure by ABA, MJ, elicitors, and even S1P (Suhita et al., 2004; Gonugunta et al., 2008). Cytosolic alkalization and production of NO in the guard cells and stomatal closure were observed on exposure to ethephon (source of ethylene) and pyrabactin (Jing et al., 2010; Puli and Raghavendra, 2012). Similarly, darkness or ultraviolet B (UV-B) exogenous Ca^2+^ induced stomatal closure was also accompanied by the increase in cytoplasmic pH and ROS (Ma et al., 2013; Zhu et al., 2014). In a reverse of the situation, fusicoccin (a fungal phytotoxin, produced by *Fusicoccum amygdale*) induced stomatal opening, by causing cytoplasmic acidification, and lowering of NO levels, even in presence of ABA (Huang et al., 2013).

Among the upstream components leading to the alkalization of cytoplasm in guard cells are the elevated ROS, PA/PLD, NO, and S1P/phytoS1P. However, the exact trigger of guard cell alkalization on exposure to ABA or MJ or elicitors and the downstream events of cytoplasmic pH change are not clear. A possibility is that on cytoplasmic alkalization, the ${\text{K}}_{\text{out}}^{+}$ channels are activated, triggering K^+^ efflux and collapse of turgor in guard cells (Blatt and Armstrong, 1993). Cytosolic alkalization needs to coordinate with the increase in cytosolic free Ca^2+^ during ABA or MJ induced stomatal closure (Islam et al., 2010). Unlike the role of ROS, NO, and cytosolic Ca^2+^as points of convergence and divergence, the action of cytoplasmic pH seems to be parallel. Further experiments are needed to make cytoplasmic pH qualified to be called as a point of convergence.

#### G-proteins

Although the modulation of heterotrimeric G proteins is known to be an important component leading to stomatal closure, the exact mode of G-protein action is ambiguous. Ge et al. (2015) suggested that ethylene induced stomatal closure was mediated through Gα induced ROS production in *Arabidopsis thaliana*. In similar case, Arabidopsis *gpa1* mutants, deficient in G-protein α subunit, are impaired in Ca^2+^-channel activation, and ROS production, in response to ABA (Zhang et al., 2011). G-proteins were essential for the production of ROS as well as NO during the effects of UV-B irradiation or external Ca^2+^ (Li et al., 2009; Zhang et al., 2012; He et al., 2013). Most of these evidences suggest that G-proteins induce an increase in the levels of ROS in guard cells. It is not clear if ROS production is due to or independent of NADPH oxidase.

#### Phospho- and sphingolipids

Phosphatidic acid (PA), the product of phospholipase C/D (PLC/PLD) induced stomatal closure by inhibiting ${\text{K}}_{\text{in}}^{+}$channel in the guard cells, besides interacting with ABI1 and activating NADPH oxidase (Jacob et al., 1999; Zhang et al., 2004). NO induced stomatal closure was restricted by PLC/PLD inhibitors (Distéfano et al., 2008), suggesting that PA acts downstream of the NO during stomatal closure in *V. faba.* Furthermore, ABA-induced NO production was impaired in *pld*α*1* mutant guard cells (Distéfano et al., 2008). Phosphoshingolipids such as sphingosine-1-phosphate (S1P) and phytosphingosine-1-phosphate (phytoS1P) regulate multiple functions in plants besides stomatal closure (Ng et al., 2001; Coursol et al., 2005; Puli et al., 2016). ABA activates sphingosine kinases (SHPKs), leading to the production of S1P. However, our knowledge of downstream signaling components of S1P is limited (Coursol et al., 2003).

### Interactions among signaling components and with environmental factors

Signaling components, particularly ROS and NO, play an important role in not only stomatal closure but also in integrating stimuli from abiotic or biotic stress (Song et al., 2014; Saxena et al., 2016). The marked interactions between ROS, NO, Ca^2+^, and pH are pointed out (Zhang et al., 2001; Gonugunta et al., 2009; Song et al., 2014). ROS and NO interact with each other and can increase cytosolic Ca^2+^ and modulate ion channels. However, the feedback relationship between NO and ROS is obscure. Similarly, cytoplasmic pH may act directly on ion channels, particularly ${\text{K}}_{\text{out}}^{+}$ or indirectly by modulating ROS and/or NO, yet the mechanism of such action is not completely clear. Further, Ca^2+^ also can interact with NO and pH (Wang et al., 2011; Gayatri et al., 2013). It is likely that ABA plays a key role in these interactions. Endogenous ABA is involved during MJ-induced stomatal closure (Munemasa et al., 2007, 2011; Ye et al., 2013). Both the Ca^2+^-dependent and Ca^2+^-independent signaling pathways are considered to function during stomatal closure (Kim et al., 2010; Roelfsema et al., 2012). However, the interrelationships of such Ca^2+^-dependent and independent pathways during guard cell signal transduction are yet to be elucidated.

Interactions of guard cell signaling components with environmental factors are not only interesting but are essential for adaptation. Drought raises the levels of ROS and ABA levels in plant tissues, with both these phenomena leading to stomatal closure (Saxena et al., 2016). The effects of CO_2_ induced stomatal closure can also be mediated by ABA (Chater et al., 2015). Further experiments are needed to identify the exact link between CO_2_ and ABA. An increase in ROS due to elevated CO2 in guard cells (Kolla et al., 2007) could raise the endogenous ABA levels and amplify the signaling events leading to stomatal closure. Similar involvement and interactions of ROS, NO, and pH are reported during UV-B induced stomatal closure (He et al., 2013; Zhu et al., 2014).

## Concluding remarks

The patterns and action sequence of signaling components during stomatal closure have been worked out using different triggers, such as ABA, MJ, and chitosan (Gonugunta et al., 2009). Both plant hormones or microbial elicitors cause an increase in ROS, NO, pH, and free Ca^2+^ of guard cells, modulate ion channels, and cause an efflux of K^+^/Cl^−^/malate from guard cells, leading to stomatal closure. We emphasize that ROS, cytosolic Ca^2+^, and ion channels are the points of convergence (Figure 1). The cytosolic pH, G-proteins, and phospho-/sphingolipids are also important components during stomatal closure but they may be acting in parallel. Further work required to elucidate the perception of signals, such as methyl jasmonate or elicitors and how they activate NADPH oxidase leading to ROS production. Several of the unresolved questions make the stomatal guard cells an ideal system for studying signal transduction mechanism in plant cells.

## Author contributions

AR proposed the topic. AR and AS collected the literature, critically assessed the information, and wrote the manuscript together.

### Conflict of interest statement

The authors declare that the research was conducted in the absence of any commercial or financial relationships that could be construed as a potential conflict of interest.
